# Development of user-customized online teaching technology based on GPT, enhanced by generative AI-based motion recognition

**DOI:** 10.1038/s41598-025-30469-5

**Published:** 2025-12-01

**Authors:** Kyoung-Geun Cho, Zahra-Batool Jaffrey, Hun-Hee Cho, Jun-Woo Lee, Ye-Jin Lee, Seon Uck Paek, Seo-Young Won, Zolzaya Dashdorj, Erdenebaatar Altangerel, Tae-Koo Kang

**Affiliations:** 1 Department of Human Intelligence Robot Engineering, Sangmyung University, Cheonan, Republic of Korea; 2 TDI Co., Seoul, Republic of Korea; 3 Computer Science Department, Mongolian University of Science and Technology, Ulaanbaatar, Mongolia; 4 Department of Software, Sangmyung University, Cheonan, Republic of Korea

**Keywords:** Motion recognition, Generative AI, GPT, Teaching system, Health care, Engineering

## Abstract

This paper addresses current issues in existing joint tracking and motion recognition algorithms for Human Pose Estimation. It proposes a solution using the Numerical Discriminator Generative Adversarial Network (ND_GAN) to improve the performance of vision-based motion recognition technology. Existing algorithms face challenges in accurately tracking joints in crowded spaces or with users wearing special attire, resulting in reduced accuracy and inconsistent results. The proposed ND_GAN consists of three integrated modules, enabling more precise joint estimation even in complex environments. Experiments were conducted using yoga videos from Hanchoom, a home training platform by TDI, comparing the performance of MediaPipe and ND_GAN on both original and leg-masked videos. Quantitative evaluation showed that ND_GAN achieved 94.2% PCK@0.5, 93.1% PCK@0.2, and an F1-score of 92.8%, marking an improvement of over 30% compared to the existing MediaPipe model. Notably, ND_GAN consistently estimated joint coordinates even in occluded video conditions, demonstrating significant performance gains in scenarios where traditional models failed. Furthermore, ND_GAN outperformed cutting-edge state-of-the-art models such as DWPose and ViTPose by more than 15% in accuracy, confirming its robustness in real-world environments with various occlusion conditions. Additionally, a teaching system using GPT is introduced to provide real-time feedback on movement errors, supporting users in effectively learning the motions. This integrated approach marks a significant advancement in addressing Human Pose Estimation complexities, enhancing overall vision-based motion recognition efficacy.

## Introduction

### Research background

Computer vision aims to implement human visual perception abilities in computers. Since the outbreak of COVID-19 in 2019, the emphasis on non-face-to-face interactions has increased, shedding more light on the development of computer vision and artificial intelligence technologies.

**Fig. 1:**
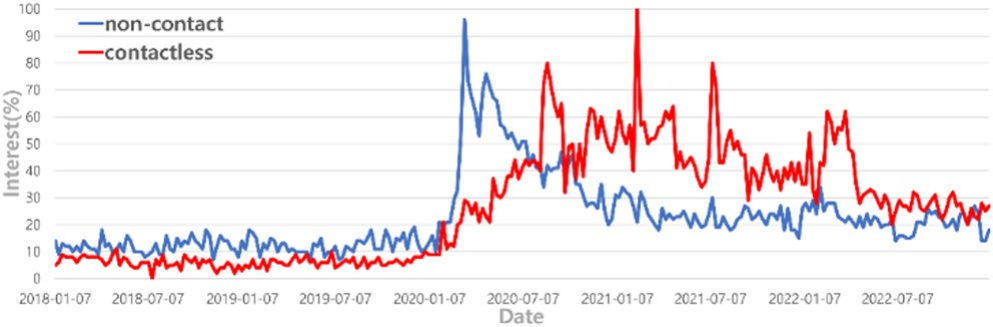
A change in interest about Google Trends non-contact and contactless.

Figure 1 depicts the change in keyword searches using the Google search engine. As shown in Fig. 1, the keywords ‘non-contact’ and ‘contactless’ started gaining attention due to concerns about virus transmission through face-to-face interactions. With the onset of non-face-to-face environments, the consumption of in-person services provided by individuals such as healthcare trainers or choreographers significantly decreased after the emergence of COVID-19, leading to an increase in the proportion of non-face-to-face service consumption^1^.

In response to this shift, society has actively developed computer vision and artificial intelligence to enable individuals to use services that traditionally required face-to-face interactions in front of mechanical elements like computers. In the context of health care services, trainers aim to observe and assess the precise movements and postures of individuals receiving health care services in face-to-face settings. They provide feedback on movement and posture issues, guiding individuals to use the services correctly. The challenge is providing such health care services in a non-face-to-face environment, which can be addressed using Human Pose Estimation, a computer vision technique that recognizes human joints in video and estimates posture. This technology is suitable for everyday services as it requires less mechanical and technical investment compared to traditional methods that involve attaching expensive equipment to individuals. Despite the resurgence of face-to-face activities, individuals who have experienced the advantages of non-face-to-face services are increasingly shifting towards receiving healthcare services through home-based care. Due to these reasons and the changing landscape of other services, the continuous development of Human Pose Estimation is ongoing, meeting the demand for objective assessment of postures and movements in service environments.

### Limitations of current motion recognition model algorithms

The described services demand the implementation of human action recognition systems using Human Pose Estimation. Open-source vision-based joint tracking AI technologies for action recognition, such as OpenPose and MediaPipe, utilizing on-device machine learning, are prominent models. In a paper titled “Surveillance and Warning System against Unauthorized Garbage Dumping Using YOLO-v5 and OpenPose.” The authors use OpenPose for joint tracking to recognize the movements of individuals littering and complete the system^2^. Similarly, in a paper titled “Dance Movement Guide System using MediaPipe,” the authors apply MediaPipe for joint tracking and use it to recognize dance movements in a human action recognition system^3^. However, the underlying assumption in both papers is that the technology can only be applied in open spaces where a person’s joints are fully visible without obstruction. The experimental conditions in both papers involve conducting experiments in open spaces without any objects, wearing ordinary clothes rather than specialized attire.

**Fig. 2:**
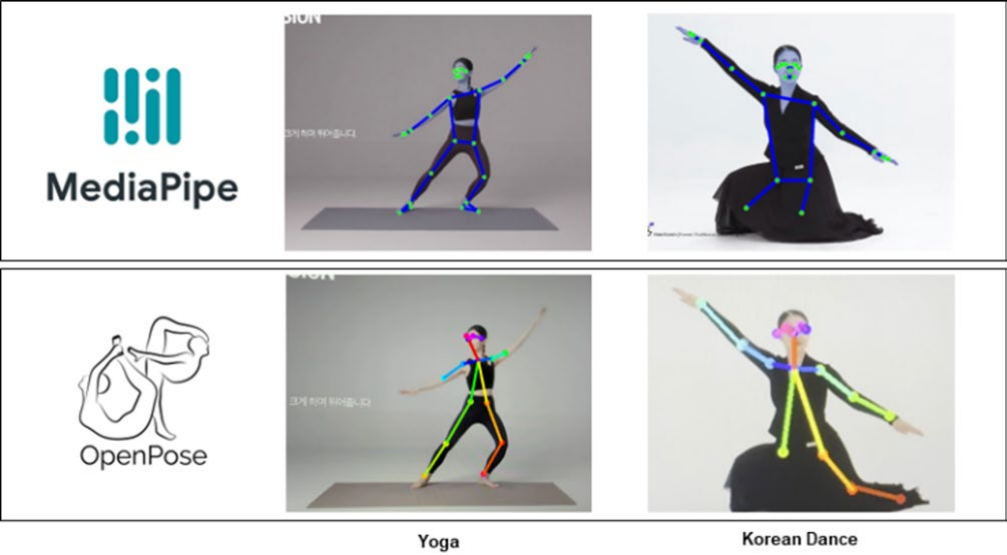
Pose estimation under clothing occlusion for each method.

If the experiment environment is changed to apply vision-based joint tracking AI technology to individuals whose joints are partially obscured by obstacles or individuals wearing special clothing used in modern or traditional dance, the recognition rate becomes inconsistent due to the nature of computer vision, as depicted in Fig. 2^4,5^.

### Significance of the research

Since the COVID-19 pandemic, demand for non-face-to-face services has surged, making technologies that accurately recognize and provide feedback on user movements more important than ever in fields such as online education, home training, and remote rehabilitation. Human Pose Estimation, the core technology behind these services, tracks users’ joint positions in real time to analyze their movements. However, widely used joint-tracking algorithms such as OpenPose and MediaPipe suffer from the inherent limitations of computer vision—under changing lighting conditions, complex backgrounds, varied clothing, joint occlusion, or unusual attire, their detection rates become unstable and accuracy drops sharply. Recent pose-estimation studies like DWPose^6^ and ViTPose^7^ have been able to mitigate occlusion to some extent, but they still exhibit unstable accuracy under heavy occlusion. This not only degrades service quality but also negatively impacts the user experience. The main contributions of this study are as follows:

1. Temporal frame management and problem-frame identification:
  We construct an Image Bank to efficiently manage metadata across consecutive frames, enabling rapid identification of frames where joints become occluded or anomalies occur during real-time streaming. By pinpointing timestamps at which conventional algorithms fail, we determine when correction is necessary in subsequent stages.
2. Numerical GAN–based joint coordinate generation module:
  When visual features around an occluded joint are lost, our generation module predicts the most statistically likely joint coordinates based on surrounding information (neighboring frame joint locations and a human kinematic model). This module adopts a GAN architecture optimized numerically (Numerical Discriminator), generating “coordinates” directly rather than simply restoring an image. By learning the underlying distribution, it flexibly handles cases of unseen unusual clothing or lighting changes without explicit prior training.

(3) Discriminator module for coordinate verification and dynamic replacement:
  The discriminator evaluates in real time whether the generated joint coordinates are plausible. If they fall below a validity threshold, the system replaces them using results from an existing model (e.g., MediaPipe) or adjacent-frame information. This immediate verification and correction mechanism ensures that, even if a single module’s generated coordinates are distorted, the final joint positions provided to users remain stable and trustworthy.

Through these three contributions, ND_GAN can estimate joint positions more accurately and robustly than existing methods in real-world environments with unpredictable factors such as lighting changes, complex backgrounds, varied clothing, and partial occlusion.

**Fig. 3:**
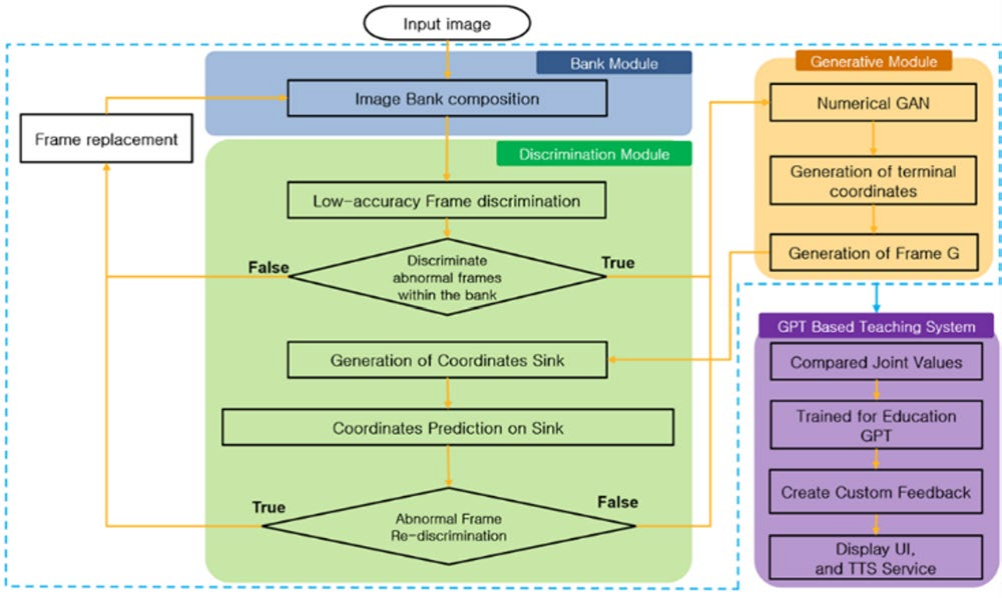
Research model algorithms overview.

## Enhancing motion recognition with generative AI

### Research model algorithms overview

The overview of the research model in this paper is depicted in the following figure.

The overview of the research model in Fig. 3 consists of three modules: the Bank Module, the Generation Module, and the Discrimination Module. These three modules interact to enhance the performance of action recognition. Images sent from the Bank Module undergo accuracy assessment in the Discrimination Module before moving to the Generation Module. Utilizing Numerical GAN in the Generation Module, joint coordinates are generated. The generated coordinates are then passed back to the Discrimination Module, where a sink mechanism is employed to comprehend the context of the frames. After prediction/generation and accuracy assessment, the Discrimination Module replaces the original image with the generated one. This entire flow, involving joint tracking based on the replaced discrimination information, illustrates the overall process of action recognition. The algorithm encompassing these modules is defined in this paper as Numerical Discriminator Generative Adversarial Network (ND_GAN).

Additionally, implementing an educational system trained on GPT-based algorithms, utilizing a completed algorithm to compare user actions with image actions, and providing feedback to users through UI and TTS services, creates a teaching system.

### Bank module for image storage

#### Interaction of bank module

**Fig. 4:**
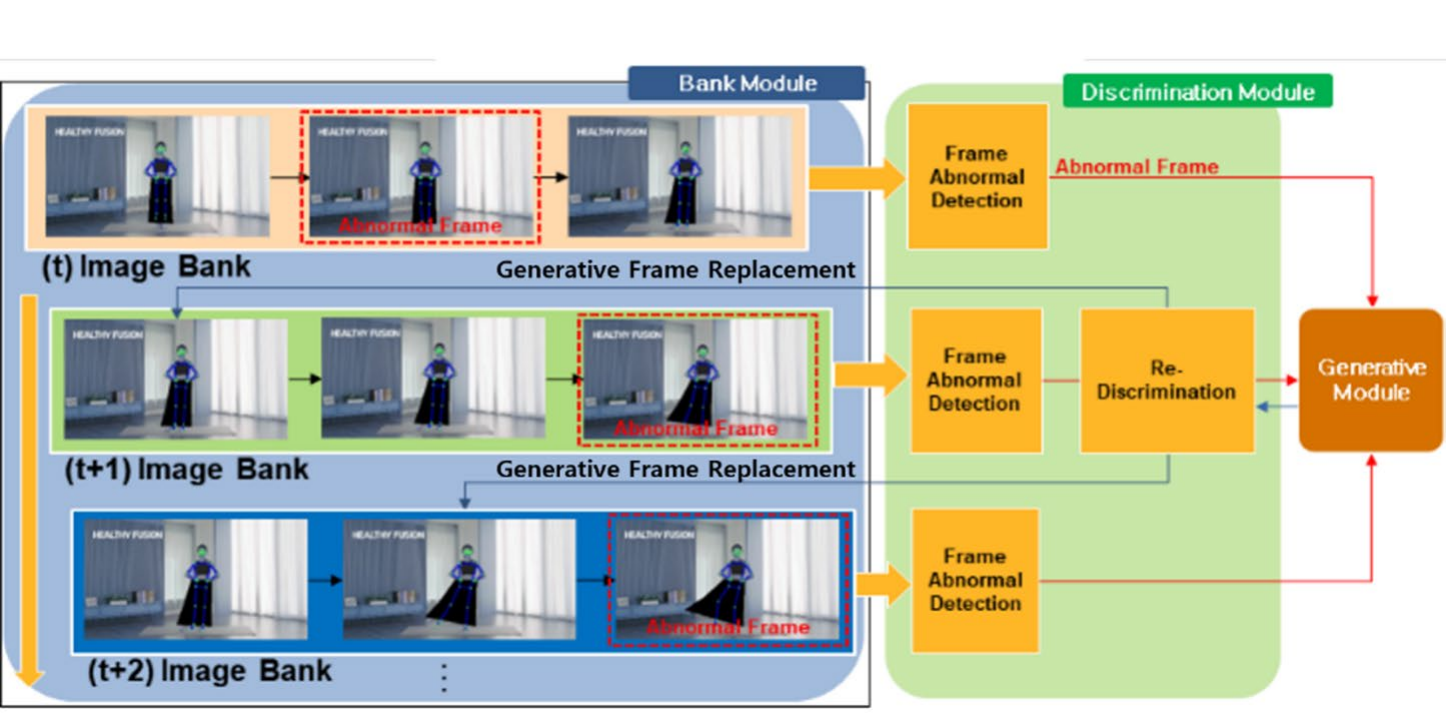
Interaction of bank module (*N* = 3).

Figure 4 illustrates the interaction of the Bank Module. In the figure, the Bank Module applies MediaPipe Pose to video frames and sends them to the Discrimination Module for assessment. The generated joint coordinates in the Generation Module are then passed through discrimination once again to replace the image in the Bank Module. This allows the Bank Module to sequentially replace frames, enhancing the accuracy of the output frames compared to the accuracy of frames before using ND_GAN.

#### Structure of bank module

In the Bank Module, images in the form of Image Banks, containing N frames, are sequentially stored over time. The sequential storage of N frames is designed to aid in the detection of abnormal frames, allowing the system to detect the next frame after an anomaly. In Fig. 4, the second or third frame of the Image Bank is detected as an abnormal frame. The detected abnormal frame undergoes repeated regeneration until it satisfies the Threshold Accuracy (TA), as illustrated in Eq. (1). By constructing such a Bank Module, the system is designed to enable accurate joint position estimation exceeding the specified Threshold value. TA is further explained in detail in section “Discrimination module for abnormal frame detection”, the Discrimination Module.

**Fig. 5:**
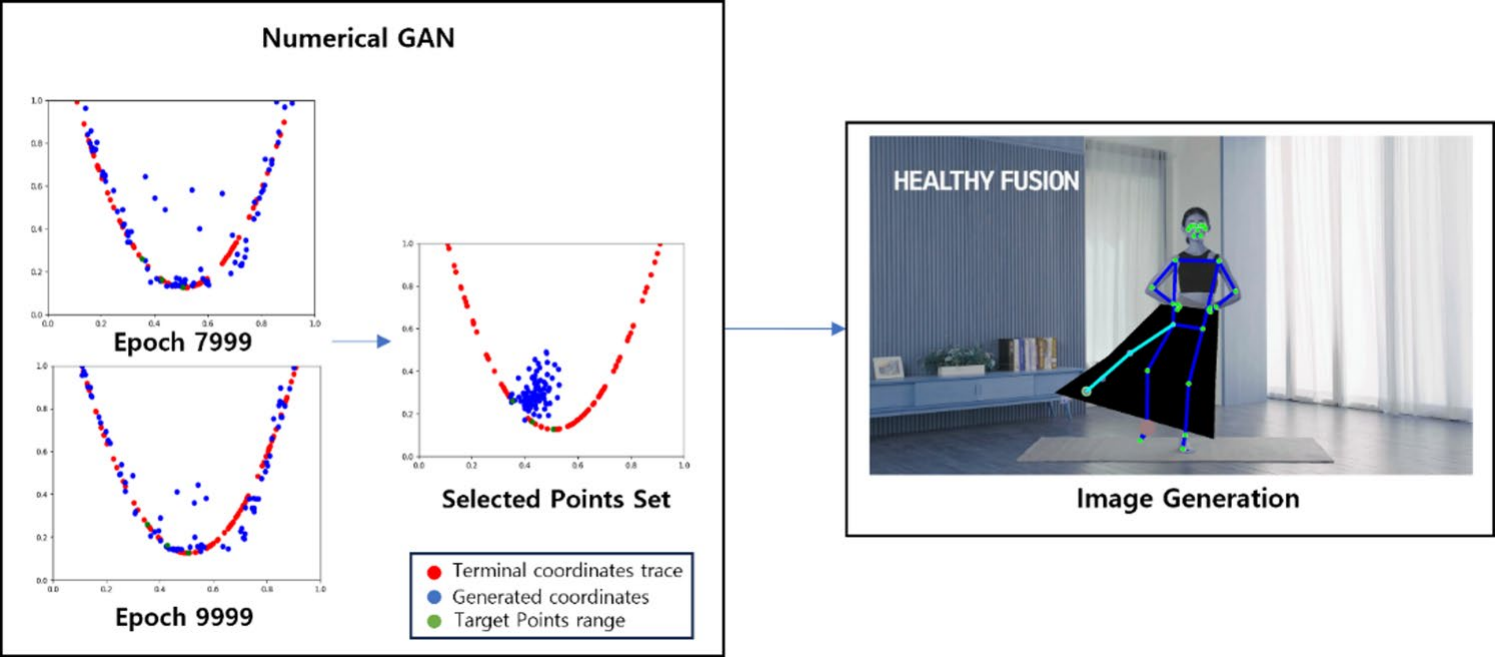
The overview of generative module.

### Generative module for image generation

#### Interaction of generative module

Figure 5 defines a total overview of the Generative Module. In Fig. 5, the red dots indicate the range of possible movement for the leg extremities in the video. The Generative Module employs a Generative Adversarial Network (GAN) to numerically generate joint coordinates based on the range of motion of human joints. Using the generated joint coordinates, the module constructs joint coordinate values through a predicted synchronized context in the Discrimination Module, ensuring that this context aligns with the joints in the frame.

#### Numerical GAN for joint coordinate generation

Table 1 defines the structure of Numerical GAN. Sample latent vectors (z) are drawn from a normal distribution and fed into the generator (G) to produce 2D joint coordinates (X_fake). The discriminator (D) is trained on real joint samples (X_real) labeled as true and generated samples (X_fake) labeled as false. With D’s weights frozen, new latent vectors (z) are used to generate coordinates, which are evaluated by D to compute a generator loss and update G. At regular intervals, D’s accuracy on real versus fake samples is measured to monitor training progress.

**Fig. 6:**
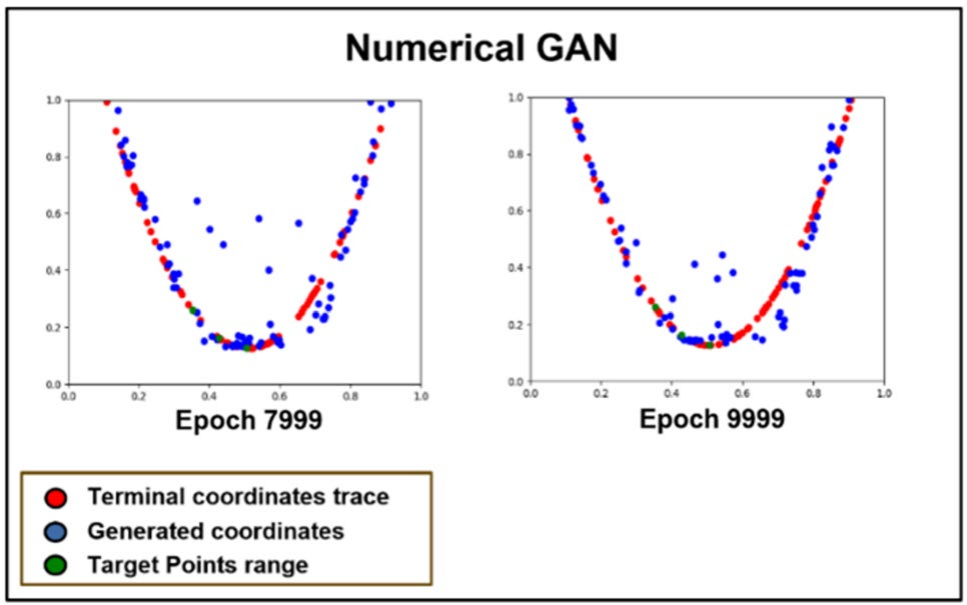
The training process of Numerical GAN.

Figure 6 depicts the process of training the Numerical GAN. For the ankle coordinates where estimation is challenging, the range of motion for leg movement is set as a quadratic curve graph. GAN is then used to generate terminal coordinates. Comparing the results after 8000 iterations, it is observed that with an additional 2000 iterations, the generated coordinates get closer to the range of terminal coordinates traced. However, the training, as shown in Fig. 6, lacks limitations on the generated coordinates, extending beyond the feasible range of human motion and producing values that cannot be utilized, leading to inefficiency in the algorithm. Recognizing this issue, the training is constrained within a valid range, as illustrated below.

**Table 1:**
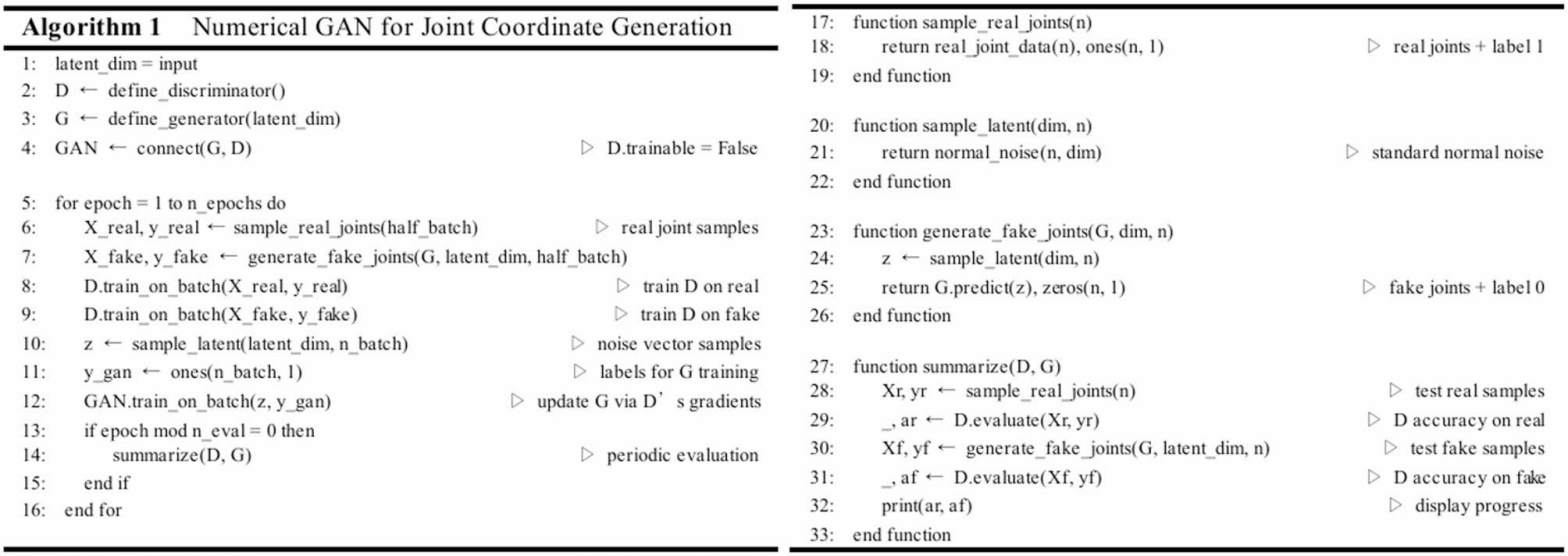
Structure of Numerical GAN.

**Fig. 7:**
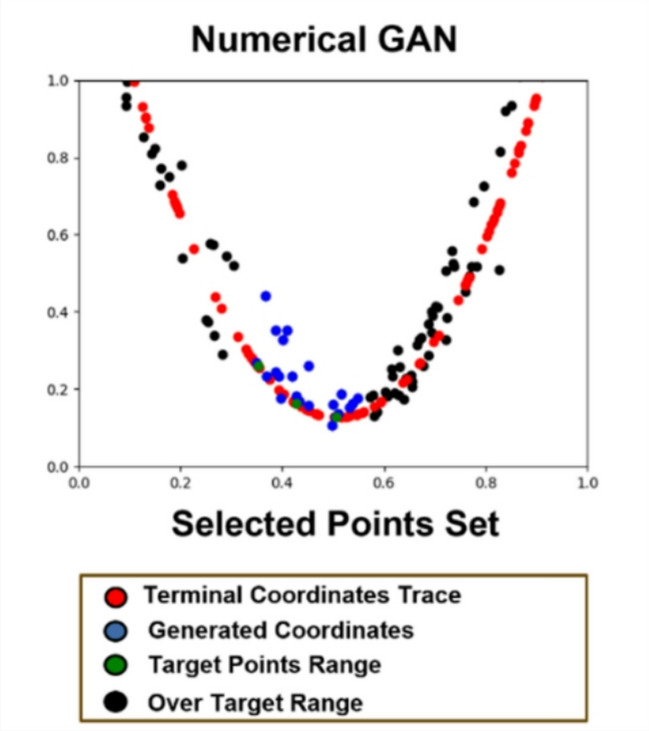
The learning process of numerical GAN after the target range is set.

Figure 7 illustrates the process of specifying the maximum and minimum points of the range of motion for terminal coordinates to train the Numerical GAN. By training in this manner, it is confirmed, as shown in Fig. 7, that Numerical GAN predominantly generates valid coordinates^8^.

#### Image generation through numerical GAN

**Fig. 8:**
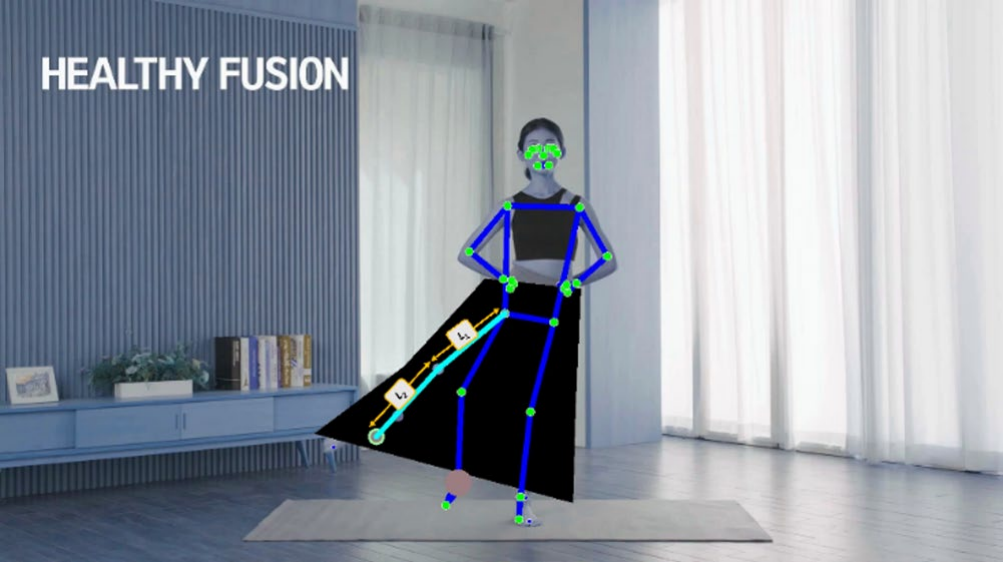
Image generation result through Numerical GAN.

The results of applying the generated coordinates within the feasible range using Numerical GAN to the frame are depicted in Fig. 8. The obtained results are selectively generated coordinates that match the frame’s synchronization through the Sink Weight Discriminator in section “Sink weight discriminator for joint position prediction” of the Discrimination Module. Without Sink Weight Discrimination, it was observed that the selected coordinates did not align with the person’s movements and joints, leading to a mismatch in the person’s actions and joints. For the estimated terminal coordinates, the inverse kinematics concept was introduced using the length values from the pelvis to the knee (L1) and from the knee to the ankle (L2) of the person’s leg. Based on the generated terminal coordinates, the inverse kinematics concept is utilized to estimate the joint shapes of the leg and complete the image generation.

### Discrimination module for abnormal frame detection

**Fig. 9:**
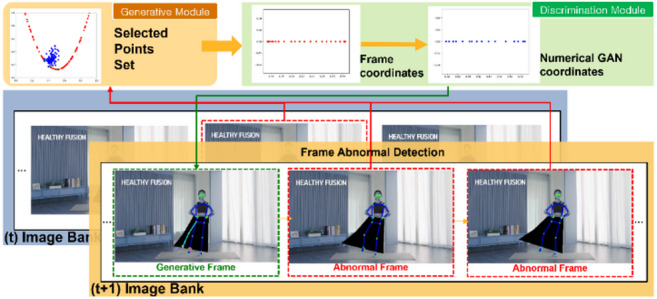
Overview of discrimination module.

**Fig. 10:**
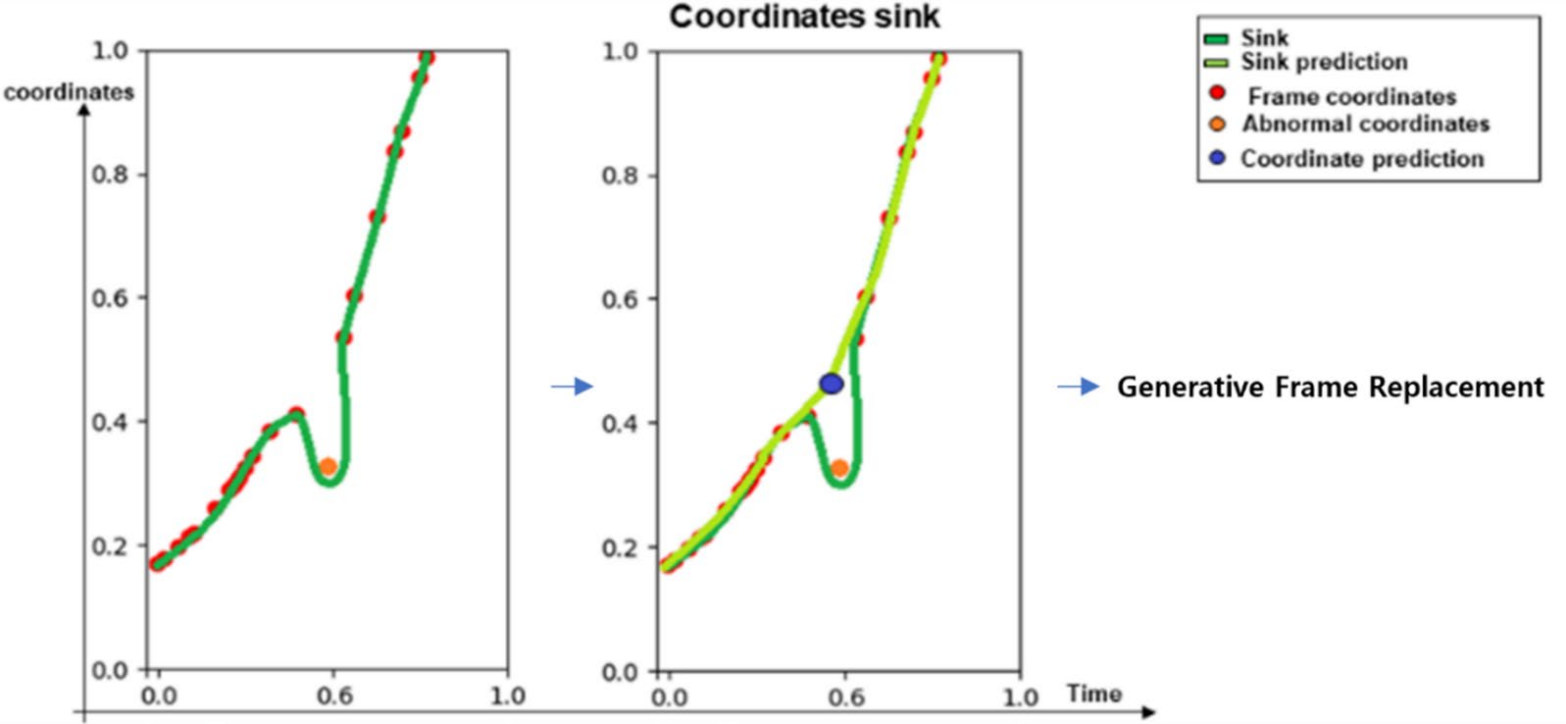
Sink weight discrimination.

Figure 9 illustrates how the Discrimination Module serves as a rigorous quality gate for every set of joint coordinates proposed by the Generative Module. When a candidate coordinate set arrives, each joint is first re-evaluated against the same confidence metrics used by MediaPipe to ensure it meets the minimum threshold. Any coordinate falling below this threshold is immediately flagged for replacement and prevented from propagating further. Those that pass the initial confidence check are then submitted to the Sink Weight Discriminator, which enforces temporal and kinematic consistency by measuring the weighted discrepancy between the generated values and the statistical distribution learned from adjacent frames. This sink‐weight regularization pulls minor outliers back onto a smooth motion trajectory, correcting subtle deviations and reinforcing realistic joint paths. If a coordinate set fails either the confidence filter or the sink‐weight stage, the Dynamic Replacement logic is triggered, substituting the unreliable estimate with a backup prediction from MediaPipe or a validated coordinate from a neighboring frame. In this way, the pipeline visualized in Fig. 10 guarantees that only joint positions that are individually plausible and globally coherent re‐enter the Image Bank for downstream processing.

#### Interaction of discrimination module

The Discrimination Module evaluates frames received from the Bank Module in units of Image Banks. Based on accuracy, it identifies frames with TA values equal to or less than Eq. (1) as abnormal frames. TA is expressed as a percentage, following the L2-norm formula, depicting accuracy based on the coordinate distance difference between the original and generated coordinate values.

1 $$TA=100 - \sqrt {{{\left( {OX - GX} \right)}^2}+{{\left( {OY - GY} \right)}^2}} *100$$

$OX$ and $OY$ are the human joint coordinates, and $GX$ and $GY$ are the coordinates generated by Numerical GAN. The identified abnormal frames undergo the generation of terminal coordinates through Numerical GAN in the Generative Module. The terminal coordinates generated are then synchronized through Sink Weight Discrimination in the Discrimination Module. Frames synchronized up to Sink undergo re-evaluation, and if the accuracy exceeds the TA value through re-discrimination, they are deemed eligible for reassignment to the next Image Bank. The threshold used in the reevaluation was set to 90%, reflecting the average of the values ​​obtained through the total training.

#### Abnormal frame detection

In the Bank Module of section “Bank module for image storage”, abnormal frames are selected based on the accuracy judgment of the ‘MediaPipe’. The accuracy value can be adjusted depending on the hardware specifications and the environment in which the algorithm is implemented. Additionally, the image results are produced based on the generated coordinate values in the Generation Module, as depicted in Fig. 8. Rediscrimination is performed based on the accuracy of the MediaPipe to determine their reassignment to the next Image Bank.

#### Sink weight discriminator for joint position prediction

In the Generative Module, an implementation of Sink Weight Discriminator is carried out to find coordinate values that synchronize among the generated terminal coordinate values through Numerical GAN. In the Image Bank of the Bank Module, abnormal frames detected are used to predict the context of the estimated coordinate values of the preceding and subsequent frames, as shown in Fig. 10. This process involves extracting the coordinate values of abnormal frames that pose issues in the flow of curves. The positions of the extracted coordinate values are corrected by replacing them with the coordinates generated by the Numerical GAN.

### GPT-based teaching system

**Fig. 11:**
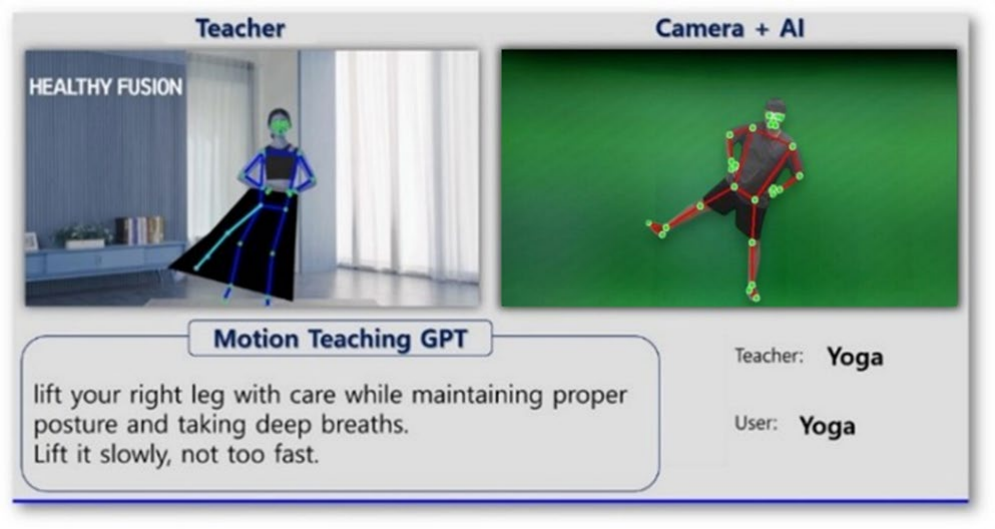
GPT-based teaching system.

Implement a teaching system using ND_GAN defined in section “Research model algorithms overview”. GPT-based teaching system is that identifies the user’s joint positions and the joint positions in the instructional videos the user is learning from, and then provides feedback to the user autonomously. This system provides a user interface (UI) that enables users to easily understand and deliver feedback to them through Text-to-Speech (TTS) technology, as shown in Fig. 11.

The educational system is designed to offer real-time posture feedback by utilizing joint values estimated by ND_GAN. When discrepancies between the user’s pose and the expert’s pose are detected, the system provides corrective feedback through TTS while simultaneously displaying the expert’s pose and the feedback in text form on the UI. This dual-mode delivery ensures that users receive immediate and accurate guidance, fostering better alignment and posture correction during the training process. By integrating GPT into the educational system, the potential of combining advanced AI technologies is realized, improving the effectiveness of non-face-to-face training and instructional services. This approach guarantees that users receive high-quality, personalized guidance, even in remote learning environments, promoting greater accessibility and efficiency in skill acquisition.

## Experimental results

### Datasets configuration

**Fig. 12:**
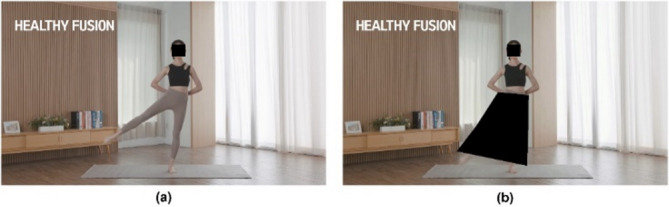
Example image used in the experiments: (a) ‘Hanchoom’ Original Video, (b) ‘Hanchoom’ yoga videos with blocked mask.

To verify whether Numerical Discriminator GAN (ND_GAN) produces normal outputs in a real video environment, an experimental setup was established. The experiment utilized yoga videos from ‘Hanchoom,’ a home training platform by TDI^9^. Figure 12 illustrates example images, including the original image and its masked image used in our experiments.

**Table 2:** Experimental environment.

	Experimental environment
Videos	‘‘Hanchoom’, the home training platform by TDI
Videos Length	15s
Training Epochs	10,000
Comparing Model	MediaPipe(Google)

As shown in Table 2, we selected the required video for the experiment and designated it as the dependent variable from the ‘Hanchoom’ home video training platform by TDI. Subsequently, an experiment was conducted by applying the existing joint estimation model to the same video after applying leg masks for performance comparison. Then, proceed with training the Numerical GAN, maintaining a consistent iteration count. Lastly, we proceed with numerical GAN training while keeping the number of iterations constant. After training is complete, we conduct experiments using a model that applies the ND_GAN algorithm (Table 3).

**Fig. 13:**
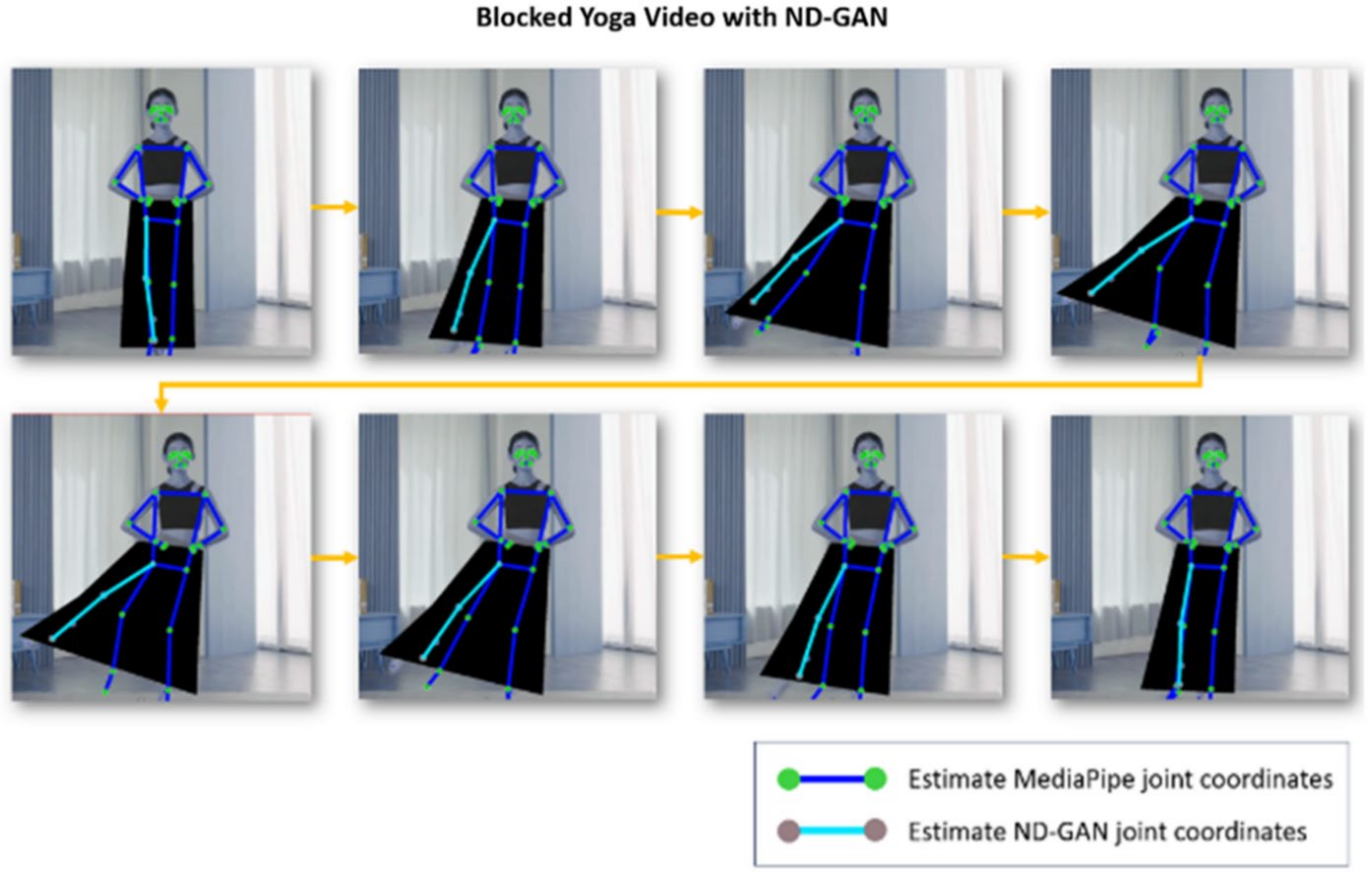
ND_GAN to blocked video result.

### Experiment results for the discriminator’s accuracy of numerical GAN

In this section, we experimented with the Discriminator’s accuracy. The experiments aimed to evaluate the performance of the Numerical Discriminator GAN (ND_GAN) in real-world video environments using yoga videos from the ‘Hanchoom’ home training platform. The experimental setup involved 15-second video clips and a training period of 10,000 epochs. A total of 100 repeated tests were conducted, incrementing the epoch by 2000 units for Numerical GAN training. The performance of the MediaPipe and ND_GAN was compared on both original and masked videos, with a primary focus on assessing how well ND_GAN could estimate joint positions under occlusion.

**Table 3:** Discriminator accuracy of numerical GAN.

Epochs	Without target range discriminator accuracy	Target range set discriminator accuracy
2000	0.209	0.68
4000	0.37	0.42
6000	0.36	0.48
8000	0.36	0.472
10,000	0.34	0.498

### Qualitative results of ND_GAN

Figure 13 illustrates the results of the qualitative evaluation. To emulate occlusion scenarios, specific joints in the ‘Hanchoom’ dataset were intentionally masked. The baseline MediaPipe model produced predictions visualized as green dots and blue lines, whereas the outputs of the proposed ND_GAN are represented by gray dots and light-blue lines.

**Fig. 14:**
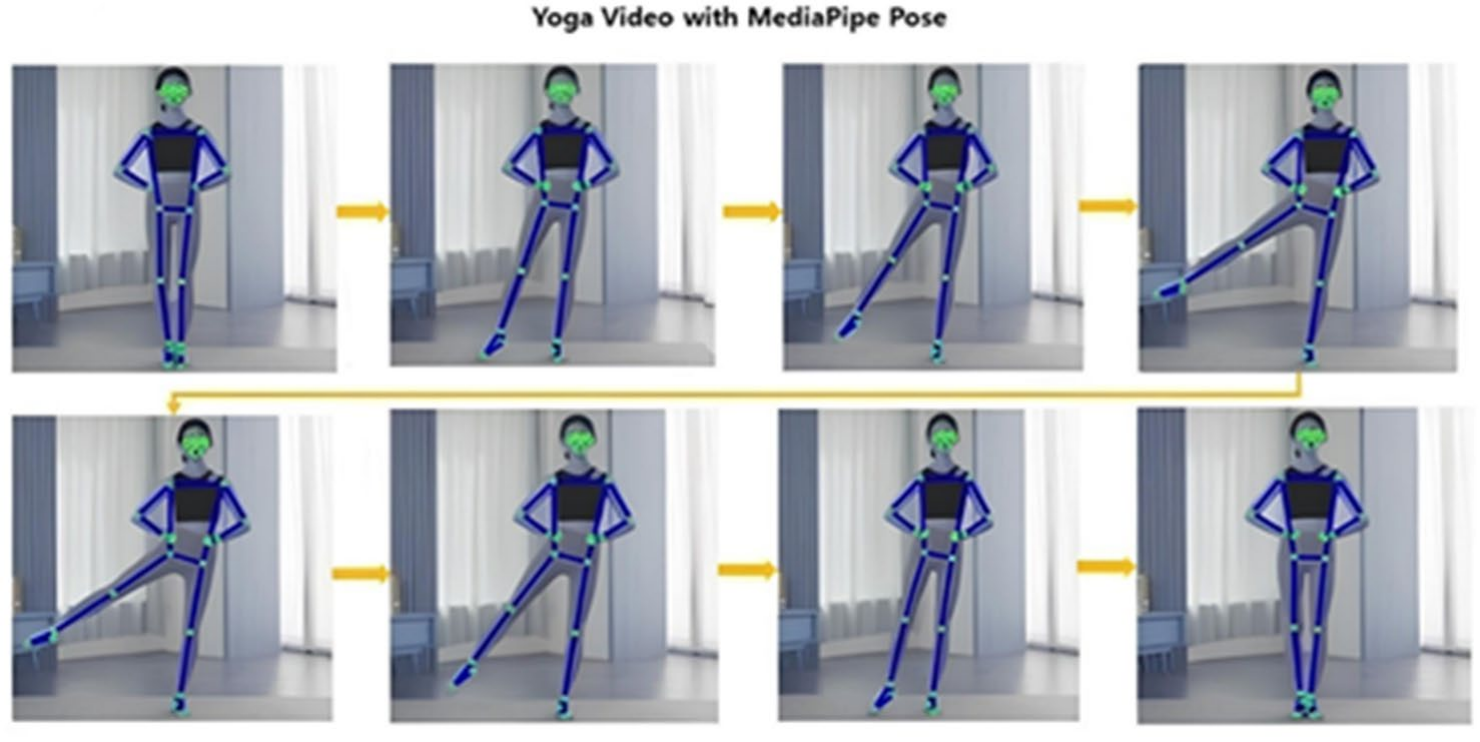
MediaPipe to original video result.

**Fig. 15:**
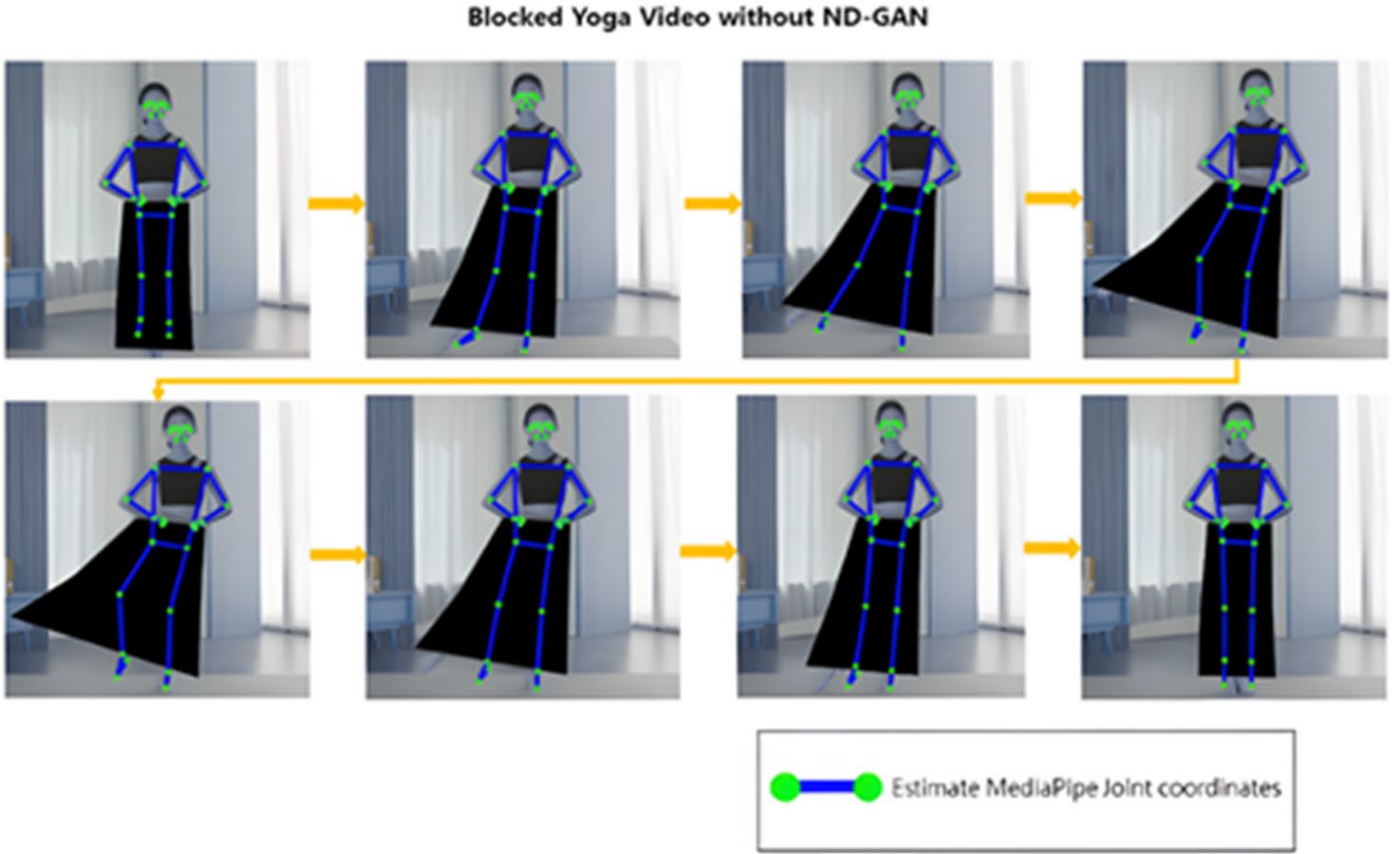
MediaPipe to blocked video result.

As observed, MediaPipe exhibited significant limitations in detecting and tracking occluded joints, frequently failing to localize them. In contrast, the proposed ND_GAN demonstrated robust performance, consistently maintaining accurate tracking of joints even under severe occlusion. These results qualitatively reinforce ND_GAN’s effectiveness in handling challenging pose estimation scenarios characterized by partial visibility.

### Quantitative results of ND_GAN

To further validate ND_GAN’s performance, we conducted comparative experiments against DWPose, a recent knowledge distillation method, and Vision Transformer (ViT) models^11^. We also included ViTPose, the current state-of-the-art model for pose estimation, known for its high accuracy and robustness across multiple benchmarks. This direct comparison was designed to evaluate ND_GAN’s effectiveness relative to the leading techniques in the field.

**Table 4:** Comparison of dwpose and SOTA methods on occlusion in the ‘Hanchoom’ dataset.

Method	Backbone	PCK@0.5	HANCHOOM PCK@0.2	F1-Score
DWPose	MobileNet^10^	65.3	60.6	65.8
ViTPose	ViT-B	69.7	65.9	79.6
Proposed method	MobileNet	94.2	93.1	92.8

Table 4 presents the performance of DWPose, ViTPose, and our ND_GAN on the ‘Hanchoom’ dataset under occlusion scenarios. We selected the ‘Hanchhom’ dataset for all experiments to provide a rigorous testbed, as it contains numerous occlusion cases that simulate real-world challenges.

The results indicate that ND_GAN outperformed the other models by approximately 15% points in accuracy, consistently maintaining reliable joint tracking even under severe occlusion. This underscores ND_GAN’s superiority for pose estimation tasks in environments where occlusion is prevalent.

Overall, these quantitative results bridge the gap between traditional methods and advanced GAN-based approaches, highlighting ND_GAN’s capability to improve joint-tracking accuracy. They suggest that ND_GAN can serve as a powerful tool for applications such as sports analytics, physical therapy, and interactive training platforms.

### Ablation study

ND_GAN was applied to a series of experimental videos to evaluate its joint-tracking capabilities, particularly under challenging conditions involving occlusion. Table 3 presents the performance comparison between the ND_GAN-enabled system and the baseline setup without ND_GAN, using the same experimental conditions described in Table 2.

**Table 5:** Performance of existing models and ND_GAN on occlusion in the ‘Hanchoom’.

MediaPipe	ND_GAN	PCK@0.5	HANCHOOM PCK@0.2	F1-Score
o	x	64.4	50.8	63.2
o	o	94.2	93.1	92.8

In this setup, applying the original MediaPipe to scenario (a) in Fig. 12 yields the results shown in Fig. 14. As illustrated, joint estimation performs well when joints are visible, and subjects wear non-obstructive clothing. However, applying MediaPipe directly to scenario (b) in Fig. 12 leads to joint-tracking failures in cases where joints are partially or fully occluded by clothing, as shown in Fig. 15. To address this limitation, we applied ND_GAN to scenario (b), producing the results shown in Fig. 13. These results demonstrate that ND_GAN can accurately estimate joint positions even in videos where lower-body tracking is deliberately made difficult. By incorporating data generated via Numerical GAN during training, the model learned to synchronize with human motion and predict joint positions with high precision. Furthermore, the system was configured to output final joint coordinates only when the accuracy, as assessed by the Judgment Module, exceeded 90%. Compared to the original MediaPipe model, ND_GAN shows substantial performance improvements in scenarios involving partial or full occlusion. This enhancement is particularly valuable in real-world video environments, such as fitness training or dance sessions, where occlusion is common. To quantitatively assess performance, we used the Percentage of Correct Keypoints (PCK) metric for joint-estimation accuracy and the F1-score to evaluate overall predictive reliability, including the reduction of false positives and false negatives. Consistent with these observations, the quantitative results on the ‘Hanchoom’ occlusion dataset are summarized in Table 5: ND_GAN improves PCK@0.5 from 64.4 to 94.2 (+ 29.8), PCK@0.2 from 50.8 to 93.1 (+ 42.3), and F1-score from 63.2 to 92.8 (+ 29.6), demonstrating its superior ability to infer hidden or obstructed joint positions. These findings highlight ND_GAN’s effectiveness in overcoming key limitations of existing pose-estimation models and delivering more robust joint-tracking performance.

## Conclusion

This paper addresses the growing demand for non-face-to-face services driven by COVID-19 by introducing the Numerical Discriminator Generative Adversarial Network (ND_GAN), a novel solution designed to overcome the limitations of existing joint tracking and motion recognition systems such as MediaPipe and OpenPose. These traditional systems often struggle with accurate joint tracking, especially in environments where joints are obscured by clothing or environmental factors, leading to decreased motion recognition performance.

In our method, ND_GAN improves joint tracking accuracy through the integration of three core components: the Bank Module, the Discriminator Module, and the Generative Module. First, the Bank Module stores images of N frames, which are analyzed by the Discriminator Module to identify abnormal frames. The Generative Module then uses a numerical GAN to generate endpoint coordinates. These coordinates are re-evaluated by the Discriminator Module to determine whether they should replace the original frames in the next bank iteration, thereby enhancing the model’s adaptability and accuracy over time.

To ensure the validity of the generated coordinates, ND_GAN constrains the range of human motion during training, resulting in improved learning accuracy. The generated coordinates are synchronized with the video’s motion using the Sink Weight Discriminator. Final outputs are only produced if the accuracy, as judged by the Judgment Module, exceeds a predefined threshold, ensuring reliable and precise joint tracking data.

Furthermore, ND_GAN is integrated into a GPT-based teaching system, providing real-time feedback on user movements via an intuitive user interface (UI) and text-to-speech (TTS) technology. This enables more accurate and immediate feedback in remote teaching environments, significantly enhancing user engagement and learning outcomes.

Experimental results demonstrate that ND_GAN achieves superior accuracy in joint tracking, even in challenging scenarios where joints are intentionally obscured. It outperforms traditional models such as MediaPipe, particularly in environments where occlusion is common. These results highlight ND_GAN’s strong potential for real-world applications, including fitness training, rehabilitation, dance instruction, low-cost computing platforms, humanoid robots, and mobile robotics.

Thanks to its lightweight design and robust performance across diverse environments, ND_GAN represents a significant advancement in human pose estimation. It effectively addresses the key limitations of current tracking and recognition algorithms and offers substantial promise for future development, especially in remote teaching and other non-face-to-face applications.

## Data Availability

The data that supports the findings of this study is available from TDI. However, restrictions apply to the availability of these data, which were used under license for the current study and so are not publicly available. Data is, however, available from the authors upon reasonable request and with permission of TDI.For data requests related to this study, please contact the official TDI website^12^.
